# Gold Nanoparticles Mediate Improved Detection of β-amyloid Aggregates by Fluorescence

**DOI:** 10.3390/nano10040690

**Published:** 2020-04-06

**Authors:** Pedro Jara-Guajardo, Pablo Cabrera, Freddy Celis, Mónica Soler, Isadora Berlanga, Nicole Parra-Muñoz, Gerardo Acosta, Fernando Albericio, Fanny Guzman, Marcelo Campos, Alejandra Alvarez, Francisco Morales-Zavala, Marcelo J Kogan

**Affiliations:** 1 Departamento de Química Farmacológica y Toxicológica, Facultad de Ciencias Químicas y Farmacéuticas, Universidad de Chile, Santiago 8380494, Chile; pedro.jaraguajardo@gmail.com (P.J.-G.); pablo.cabrera@ug.uchile.cl (P.C.); 2 Advanced Center for Chronic Diseases (ACCDiS), Sergio Livingstone 1007, Independencia, Santiago 8380494, Chile; 3 Laboratorio de Procesos Fotónicos y Electroquímicos, Universidad de Playa Ancha, Playa Ancha 850, Valparaíso, Chile; freddy.celis.b@gmail.com; 4 Departamento de Ingeniería Química, Biotecnología y Materiales, Facultad de Ciencias Físicas y Matemáticas, Universidad de Chile, Beaucheff 851, Santiago 8380494, Chile; m.soler.jauma@gmail.com (M.S.); isadora.berlanga@ing.uchile.cl (I.B.); nparra@ing.uchile.cl (N.P.-M.); 5 CIBER-BBN, Networking Centre on Bioengineering, Biomaterials and Nanomedicine & Department of Organic Chemistry, Marti i Franques 1-11, University of Barcelona (UB), 08028 Barcelona, Spain; gerardoacosta@ub.edu (G.A.); albericio@ub.edu (F.A.); 6 School of Chemistry & Physics, University of KwaZulu-Natal, Durban 4001, South Africa; 7 Núcleo de Biotecnología Curauma (NBC), Pontificia Universidad Católica de Valparaíso, Valparaíso 2460355, Chile; fanny.guzman@pucv.cl; 8 Department of Chemistry, Faculty of Sciences, University of Chile, POBox 653, Santiago 8380494, Chile; facien05@uchile.cl; 9 Facultad de Ciencias Biológicas, Pontificia Universidad Católica de Chile, Alameda 340, Santiago 8331010, Chile; aalvarez@bio.puc.cl; 10 Centro de envejecimiento y regeneración (CARE), Facultad de Ciencias Biológicas, Pontificia Universidad Católica de Chile, Santiago 8380494, Chile

**Keywords:** gold nanorods, CRANAD-2, amyloid beta peptide, SEF, Alzheimer´s disease

## Abstract

The early detection of the amyloid beta peptide aggregates involved in Alzheimer’s disease is crucial to test new potential treatments. In this research, we improved the detection of amyloid beta peptide aggregates in vitro and ex vivo by fluorescence combining the use of CRANAD-2 and gold nanorods (GNRs) by the surface enhancement fluorescence effect. We synthetized GNRs and modified their surface with HS-PEG-OMe and HS-PEG-COOH and functionalized them with the D1 peptide, which has the capability to selectively bind to amyloid beta peptide. For an in vitro detection of amyloid beta peptide, we co-incubated amyloid beta peptide aggregates with the probe CRANAD-2 and GNR-PEG-D1 observing an increase in the intensity of the fluorescence signal attributed to surface enhancement fluorescence. Furthermore, the surface enhancement fluorescence effect was observed in brain slices of transgenic mice with Alzheimer´s disease co-incubated with CRANAD-2 and GNR-PEG-D1. An increase in the fluorescence signal was observed allowing the detection of aggregates that cannot be detected with the single use of CRANAD-2. Gold nanoparticles allowed an improvement in the detection of the amyloid aggregated by fluorescence in vitro and ex vivo.

## 1. Introduction

Alzheimer’s disease (AD) is the most common form of dementia that affects elderly people. AD is a disease that compromises memory, cognitive abilities and judgment, and it is caused by the dysfunction and loss of synapses and neurons. Although much is known about the pathological mechanisms involved in AD, there is currently no cure or effective palliative treatment. One of the most important limitations that have become increasingly evident for the development of effective therapies is that it is necessary to detect AD early before the damage occurs [1,2]. The accumulation of the amyloid beta peptide (Aβ) produced by proteolytic cleavages from the amyloid precursor protein (APP) generates toxic aggregates that are a key factor involved in the dysfunction and loss of synapses and neurons, causing AD symptoms [3,4]. Therefore, it is also necessary to develop new techniques to detect Aβ brain accumulation in order for an early diagnose of the apparition of this disease and its progress, and to also monitor the effectiveness of new therapies. Imaging techniques, such as positron emission tomography (PET) and single photon emission computed tomography, are used to study functional changes in the brain and, in the case of PET, also to detect the presence of amyloids in the brain [5]. For molecular imaging, new radiotracers, such as the Pittsburg compound B that exhibit affinity for Aβ, have been used [5]. However, this compound contains radioactive ^11^C, which is an isotope with low half-life that requires the use of a synchrotron located near the place in which the diagnosis is performed. Thus, because of the high cost associated with the use and implementation of these diagnostic techniques, which is due to the limited accessibility of equipment and the low half-life of the radiotracer, it has been difficult to use them for clinical diagnosis of Alzheimer’s disorder. For now, AD is still mainly diagnosed only when clinical symptoms appear, that is, after the disease onset. However, by the time plaques are detected, neurodegeneration has already occurred and, at this stage of the disease, existing therapies and treatments are not effective. Therefore, it is necessary to develop new inexpensive imaging methodologies with a high sensitivity and resolution, such as fluorescence imaging [6,7].

**Figure 1:**
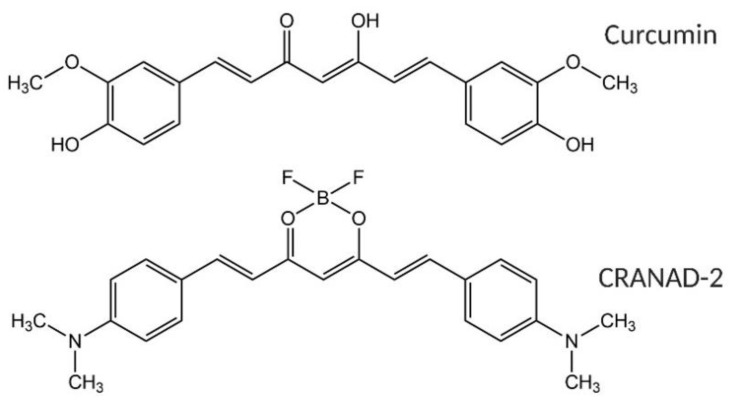
(Top) Structure of curcumin and (bottom) structure of CRANAD-2.

Currently, efforts have been focused on the early detection of AD based on fluorescence imaging. A fluorescent probe (CRANAD-2), derived from curcumin that is a natural polyphenol extracted from the rhizome of *Curcuma longa* (turmeric), was developed for this purpose (Figure 1). CRANAD-2 possesses a high affinity for insoluble amyloids aggregates, such as Aβ fibrils (Kd = 38 nM). This compound forms a complex with Aβ fibrils that exhibits a maximum emission fluorescent band centered at the near-infrared (NIR) region at 715 nm [8]. This characteristic makes this probe suitable for in vivo usage, due to the transparency of tissues to radiation from this spectral region [8]. Despite these desirable characteristics, CRANAD-2 is difficult to be detected due to its low quantum yield (20%) [8]. This issue limits its application for the detection of amyloid aggregates involved in AD. Therefore, increasing the fluorescent emission of this probe is paramount.

Gold nanoparticles (GNPs) exhibit interesting physicochemical properties, derived from their optical properties, size, and shape. Due to that, its superficial electrons can resonate in response to an incident light, a property called Surface Plasmon Resonance (SPR). GNPs can absorb light energy and efficiently dissipate it as heat as well as producing light scattering [9]. Importantly, the scattered light can enhance the fluorescent emission of fluorescent molecules located near, at an optimal distance, to the gold surface. The enhancement of fluorescence by surfaces, which is called surface enhanced fluorescence (SEF), by using plasmon nanoparticles for in vitro detection of biomarkers has been widely described [10]; recently spherical GNPs and the Rose Bengal dye have been used for the detection of Aβ aggregates [11]. However, this last system is not optimal for in vivo applications because the emission of fluorescence is in the visible region and not in the NIR where the tissues exhibit a low absorption of light.

**Figure 2:**
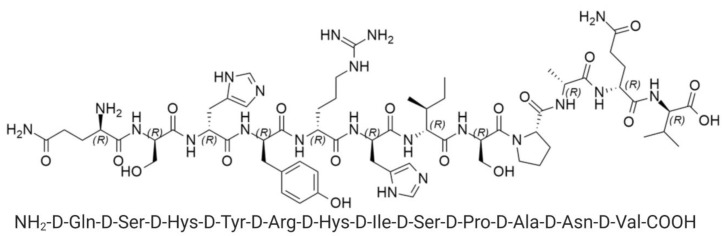
Structure of D1 peptide.

In order to improve the detection of Aβ, we used gold nanorods (GNRs) in this study, that exhibit an absorption plasmon band centered in the NIR region, to increase the fluorescence of CRANAD-2 [8]. Previously, we developed a Drug Delivery System based on GNRs functionalized with a beta sheet breaker peptide, the D1 peptide (Figure 2). D1 peptide recognizes and attaches to the Aβ fibrils (with a Kd in the sub micromolar range) targeting the GNRs to brain Aβ aggregates. D1 peptide has the capacity to inhibit the Aβ peptide aggregation and to promote Aβ aggregates disaggregation. D1 is formed by D amino acids that are stable against endogenous proteases [12,13,14].

**Figure 3:**
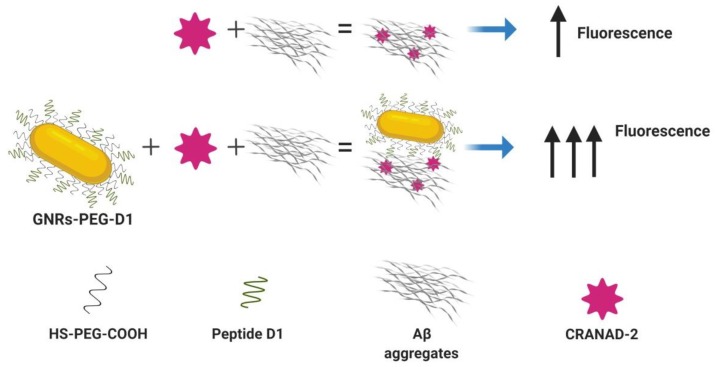
Gold nanorods (GNRs) functionalized with the peptide D1 allow the enhancement of a fluorescence signal of CRANAD-2 associated with amyloid beta peptide (Aβ) aggregates.

In the present research, we demonstrated that GNRs functionalized with the peptide D1 containing a spacer of polyethyleneglycol (PEG) (GNR-PEG-D1) promotes the enhancement of the fluorescence signal of CRANAD-2 associated with Aβ aggregates (Figure 3). The functionalized nanoparticles were characterized by different techniques: UV–vis-NIR, Dynamic Light Scattering (DLS), Electron Microscopy, and Surface-enhanced Raman Scattering (SERS), allowing us to determine the orientation and organization of the D1 molecules on the gold surface [15]. This information is crucial to understand the interaction of GNR-PEG-D1 with Aβ and the increases in the fluorescence intensity of CRANAD-2. To the best of our knowledge, this is the first time that SEF enhancement using GNPs has been evaluated for an in vitro and ex vivo detection of Aβ aggregates.

## 2. Materials and Methods 

### 2.1. Synthesis of GNRs

For the preparation of a seed solution of GNPs, a cold-prepared sodium borohydride solution (600 µL, 0.01 M) was added to 250 µL of 0.01 M HAuCl_4_ in 9.75 mL of 0.1 M cetyltrimethylammonium bromide (CTAB) in a flask, under vigorous magnetic stirring. The seed solution was kept at 27 °C for 2 h, before use. After that, 55 µL of 0.1 M ascorbic acid solution (Sigma Chemical Co., St. Louis, MO, USA) was added to a growth solution containing 75 µL of 0.01 M AgNO_3_ (Sigma Chemical Co., St. Louis, MO, USA), 9.5 mL of 0.1 M CTAB, and 500 µL of 0.01 M HAuCl_4_. Further, 250 µL of 0.1 M HCl and 12 µL of the previously prepared seed solution were added. The solution was incubated for 10 min at 27 °C and then centrifuged at a 7030× *g* for 15 min. After centrifugation, the supernatant was removed and the pellet was resuspended in milli-Q water [16,17,18].

### 2.2. GNRs PEGylation and Conjugation with D1

In the first step, the GNRs were conjugated with asymmetrical PEGs that have a thiol group (SH) at one end, and a methoxy (HS-PEG-OMe MW 5K, JenKem Technology, TX, USA) or a carboxylic acid group (HS-PEG-COOH MW 5K, JenKem Technology, TX, USA) at the other. A total of 50 μL of 1 mM HS-PEG-OMe in a water solution was added to 10 mL of 1 nM GNRs-CTAB and stirred for 10 min. After centrifugation at RCF of 16,100× *g* for 10 min, the pellet was resuspended in 10 mL of milli-Q water. Subsequently, 300 μL of 1 mM HS-PEG-COOH solution was added into the water solution, and the suspension obtained was stirred for one hour [19]. Further, the suspension was centrifuged at 16,100× *g* for 10 min, and the pellet was resuspended in 100 μL of 0.1 M 2-(N-morpholino)ethanesulfonic acid (MES) buffer pH 5.5. Subsequently, 0.2 mg of ethyl-3-(3-dimethylaminopropyl)-carbodiimide (EDC) and 0.5 mg of sulfo-N-hydroxysuccinimide (Sulfo-NHS) in 100 μL of MES were added and mixed for 15 min. The excess of EDC/Sulfo-NHS was subsequently removed by centrifugation at 16,100× *g* for 10 min [16]. The resulting pellet was mixed with 0.3 mg of D1 peptide, synthesized using a solid-phase synthesis protocol (SI), and dissolved in phosphate buffered saline (PBS) pH 7.4. The final solution was stirred overnight and centrifuged again the next day, at 16,100× *g* for 10 min, to remove the excess of D1. Then, the pellet was resuspended in milli-Q water and stored at 4 °C. The conjugations were performed following the protocols described in Morales-Zavala et al. [16].

### 2.3. Characterization of Functionalized GNRs

The nanoparticles obtained following the procedure described above were characterized by UV–vis-NIR absorption spectra, dynamic light scattering (DLS), zeta potential (pZ), scanning transmission electron microscope (STEM), and Raman spectrometer.

The UV–Vis-NIR absorption spectra and the size distribution were determined at 25 °C in a Perkin Elmer Lambda 25 spectrophotometer and in a Malvern Zeta sizer 3000 (Malvern Instruments, UK), respectively. A total of 1 mL of the colloidal sample was introduced in a cell with an optical path of 1 cm. To determine the size distribution of the samples, the results were retrieved from the intensity distribution values using the cumulant method [16,20]. 

The nanoparticles zeta potential (Zeta sizer 3000, Malvern Instruments, UK) measurements were performed in an aqueous solution at a moderate electrolyte concentration and repeated five times. The Smolochowski approximation was used to calculate the Z potentials from the measured electrophoretic mobility [20].

The morphology of GNR was determined using a scanning electron microscope with the electronic transmission module (STEM) FEI Inspect F50 (STEM conditions: HV: 30,000 KV, Mag: 500,000, HFW: 597 nm, WD: 4.3 mm, Spot: 3.0). For electron microscopy observations, a drop of the GNRs was deposited onto a Formvar carbon-coated copper microgrid (Ted Pella, Inc. Redding, CA, USA) and waited for the sample to dry before observation.

A Renishaw InVia Raman spectrometer was used to scan the Raman spectrum of the D1 peptide and the SERS spectrum of the GNR-PEG-D1. The micro-spectrometer was equipped with a 785 nm laser line and an electrically cooled CCD detector coupled to a Leica microscope. All spectra were recorded using a 50x objective. The spectral range was 200–2000 cm^−1^ and the number of acquisitions was set at 5, with 15 s integration time. The laser power chosen was 0.2 mW (less than 1%) in order to avoid photodecomposition. The samples were deposited on a thin sheet of gold to remove the intrinsic fluorescence. This surface was prepared by sputtering deposition of gold on a glass substrate under Argon plasma.

### 2.4. Synthesis and Characterization of CRANAD-2

Starting materials were purchased from Sigma-Aldrich-Merck Millipore and were used without further purification. The products were characterized by ^1^H-NMR spectroscopy (Multinuclear NEO Bruker Advance 400 MHz), FTIR-ATR spectroscopy (Thermo Scientific Nicolet iS10 coupled to an ATR SmartTM iTX accessory with a monol crystal diamond), UV–vis absorption spectroscopy (Avantes Avaspec–ULS2048L Starline Versatile Fiber-optic), and Emission spectroscopy (Perkin Elmer LS-55).

Synthesis of curcuminoid (5-hydroxy-1,7-bis[4-(dimethylamino) phenyl]hepta-1,4,6-trien-3-one): Acetylacetone (acac, 751 μL, 7 mmol) and boric anhydride (B_2_O_3_, 0.348 g, 5 mmol) were dissolved in EtOAc (8.0 mL). The reaction mixture was heated to 60 °C and stirred for 30 min until a white suspension was formed. Then, a solution containing 4-(dimethylamino) benzaldehyde (2.088 g, 14 mmol) and tri-tert-butyl borate (14 mmol) in EtOAc (14 mL) was added to the reaction mixture and stirred for 3 h at 60 °C. After cooling down for 10 min, a solution of n-butylamine (0.4 mL, 4 mmol) in EtOAc was added dropwise (5 drops/min), and the reaction mixture was stirred at room temperature for 24 h. At this point, a red-brown precipitate was formed corresponding to the boron complex, which was filtered and suspended in water for 3 h to break the complex. After a day, the pure ligand was filtered and dried under vacuum. Yield: 71.6%. Melting point 222–224 °C. ^1^H-NMR (400 MHz, CDCl_3_) δ (ppm): 16.33 (s, 1H); 7.60 (d, 2H, J = 16Hz); 7.46 (d, 4H, J = 8.8 Hz); 6.69 (d, 4H, J = 8.8 Hz); 6.42 (d, 2H, J = 16 Hz); 5.73(s, 1H), 3.03 ppm (s, 12H). FTIR-ATR: 2952 (Csp^2^-H), 2800 (Csp^3^-H), 1583 (C=O cetone), 1522 (C=C) cm^−1^ UV–vis CH_2_Cl_2_, λmax: 485 nm. Emission spectrum CH_2_Cl_2_, λemi: 566 nm (λexc = 485 nm) (See Supplementary Material).

Synthesis of CRANAD-2: The curcuminoid (188.1 mg, 0.52 mmol) was dissolved in CH_2_Cl_2_ (4 mL), and then, the boron trifluoride diethyl etherate BF_3_(Et_2_O)_2_ (0.2 mL, 0.744 mmol) was added. The reaction was irradiated with a microwave at high power for 5 minutes at 60 °C. The flask was cooled for 15 minutes. The product was filtered and washed with CH_2_Cl_2_ to obtain a black powder (274 mg yield 86%). Melting point analysis showed that that was decomposed at over 300 °C.^1^H-NMR (400 MHz, DMSO-d6) δ (ppm): 7.82 (d, 2H, J= 15 Hz), 7.68 (d, 4H, J= 8,6 Hz), 6.78 (m, 6H), 6.28 (s, 1H), 3.06 (s, 12H). FTIR-ATR: 3089 (Csp^2^-H), 2910 (Csp^3^-H), 1597 (C=O cetone), 1522 (C=C), 1046 (B-O), 1066 (B-F) cm^−1^. UV–vis CH_2_Cl_2_: λmax: 595 nm. Emission spectrum CH_2_Cl_2_, λemi: 657 nm (λexc = 595 nm). (See Supplementary Material).

### 2.5. In Vitro Fluorescence Assay for the Detection of Amyloid Aggregates in the Presence of GNR-PEG-D1

Aβ1-42 was purchased from r-Peptide (GA 30622, USA). To obtain mature Aβ fibrils, the aliquots of Aβ were treated with 1,1,1,3,3,3-hexafluoro-2-propanol (HFIP) for 30 min, to produce the monomeric Aβ form [16]. Aliquots were then lyophilized and resuspended in PBS. The final Aβ concentration was 10 μM. The samples were incubated for 3 days at 37 °C while mechanically shaken [16]. After three days, 25 μL of the incubated solution of Aβ was mixed with 20 μL PBS, 2.5 μL of CRANAD-2, and 2.5 µL of GNR-PEG-D1 (0.01, 0.1, 0.5, and 1 nM GNR-PEG-D1) in a Corning 384 bottom black plate 3575. The fluorescence was measured with an excitation wavelength of 640 nm and an emission wavelength of 715 nm.

### 2.6. Observation by Transmission Electron Microscopy of Amyloid Aggregates in the Presence of GNR-PEG-D1

An aliquot of Aβ (25 μL) incubated for three days was added to PBS (22.5 μL) and GNR-PEG-D1 (2.5 μL), then we let it sit. After 15 minutes, 10 μL of the mixture was separated and the drop was deposited on parafilm. In order to dye the fibrils, a grid was placed on the drop for 2 minutes, then washed twice with milli-Q water (1 minute per wash), and finally deposited for 2 minutes on a freshly prepared drop of 1% phosphotungstic acid. After the staining process, it was allowed to dry for 24 hours and then evaluated by transmission electron microscopy (TEM) Hitachi HT7700.

### 2.7. Ex Vivo Fluorescence Assay for the Detection of Amyloid Aggregates in the Presence of GNR-PEG-D1 in Brain Tissue

Fourteen animals B6C3-Tg (APPswe, PSEN1dE9) 85Dbo/Mmjax mice aged 1 year and 7 months were used (Alzheimer´s model). The mice were anesthetized with a mixture of ketamine–xylazine–acepromazine (4:4:1), perfused with filtered PBS (pH 7.2), and fixed by perfusion of approximately 50 mL of parafolmaldehyde 4% in PBS. Then, the mice brains were removed. 

Subsequently, the brains were left in fixation solution overnight (PFA 4% in PBS pH 7.2) and transferred to a maintenance solution (sucrose 30%, sodium azide 0.2% in PBS pH 7.2). Finally, the brains were cut in a cryostat (30 μm slices), obtaining a total of 41 slices which were mounted on the slides. The AD mice brain slides were incubated with CRANAD-2 0.24 mM for 5 min (200 μL of solution was added to each tissue slice). Then, the excess of dye was removed and the tissue washed with 50% ethanol, followed by absolute ethanol. Finally, the slides were incubated for 5 min with different concentrations of GNR-PEG-D1 in order to determine the optimal concentration needed. We evaluated five concentrations: 0.5, 0.1, 0.01, 0.001, and 0.0001 nM (200 μL of solution was added to the tissue). Imaging was performed with an Olympus CKX41 fluorescence microscope with a 10x objective before and after each incubation step. The fluorescence intensity was evaluated both in the presence and in the absence of GNRs in the same histological section.

### 2.8. Statistical Analysis

The in vitro fluorescence spectra were analyzed using the *t*-test to compare the baseline values with the enhanced values of each experimental conditions set.

The ex vivo fluorescence data obtained were analyzed using the image J software, while the statistical analysis was performed with the Mann–Whitney test included in the graphpad prism 6 software.

## 3. Results and Discussion

### 3.1. Synthesis and Characterization of GNR-PEG-D1

GNRs were synthesized following the seed-mediated growth method described by Nikoobakht et al. [17]. Subsequently, the GNRs were functionalized with asymmetric PEG molecules (HS-PEG-OMe and HS-PEG-COOH), which were chemisorbed through the SH function onto the surface of the gold nanoparticles. Then, the free N-amino terminal of D1 peptide was linked to the activated carboxylic group of HS-PEG-COOH. The carboxylic acids were activated by the reaction between EDC and Sulfo-NHS to form an amide bond between the peptide and the PEG chains. After the completion of this process, the functionalized GNRs were characterized using several techniques: UV–vis-NIR spectroscopy, DLS, zeta potential (pZ), STEM, and Raman spectroscopy.

**Figure 4:**
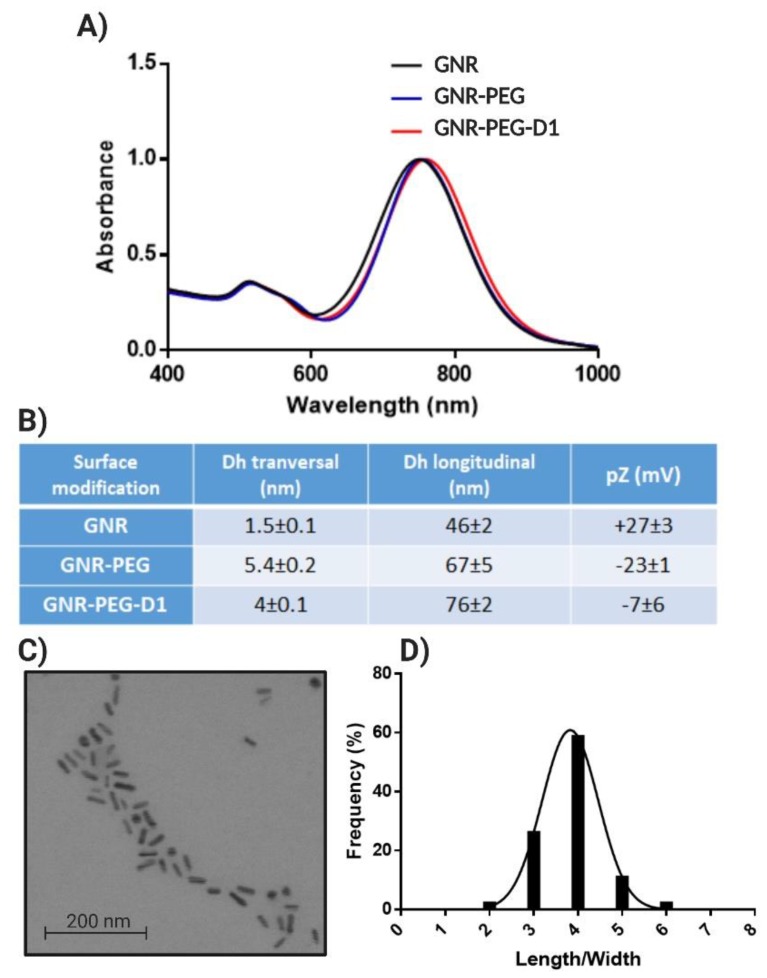
GNRs characterization: UV–vis-NIR spectra of GNRs (**A**), size distribution obtained by Dynamic Light Scattering (DLS) and zeta potential (pZ) for GNRs (**B**), STEM of GNRs (**C**), and aspect ratio length/width obtained from 500 particles (**D**).

GNRs exhibited the two characteristic bands at 520 nm (longitudinal) and 750 nm (transversal) (Figure 4A) [16,21]. The rod shape and size distribution were determined by STEM, obtaining a length of 33 ± 5 nm and a width of 8 ± 1 nm (Figure 4C), with an average aspect ratio (length/width) of 4 (Figure 4D). GNRs displayed a hydrodynamic diameter (Dh) (graphic summary in Supplementary Materials, Figure S3) of 46 nm once they were functionalized with the PEGs, which represents an increase of 20 nm in comparison to naked GNRs. Following the functionalization with the D1 peptide, the GNRs’ Dh increased by 10 nm in comparison with the GNRs-PEGs (Figure 4B). Regarding the changes of the potential zeta, it was observed that the naked GNRs (CTAB capped) had a high positive value of +27 mV, but after PEGs functionalization, a change in the pZ to a value of −23 mV was observed. After functionalization with the peptide D1, the GNRs acquired a pZ value of −7 mV (Figure 4B). It is notable that, after the chemisorption of PEG onto the surface of GNRs, the pZ changed from a positive to a negative absolute value. This result is explained by the replacement of the cationic surfactant CTAB by HS-PEG-OMe and HS-PEG-COOH. The latter contributes to the negative charge of the system due to the negative charge of the carboxyl group at pH = 7.4, belonging to HS-PEG-COOH. The pZ showed an increase of positive values in GNRs when the D1 peptide was conjugated, as was observed by Morales-Zavala et al. [16] in the process of functionalization of GNRs-Angiopep2 with D1. This is explained by the conjugation of the D1 peptide to the carboxyl groups (to form the amide bond), which contributes to the increase in the pZ of the nanoparticle due to the net charge exhibited by the peptide D1 at pH 7.4 (the isoelectric point of the peptide is 9.55). Although the final zeta potential of GNR-PEG-D1 is low in absolute value (−7 mV), the colloidal stability of this nanosystem can be attributed to steric effects produced by the functionalization with PEG chains on the gold surface and the further conjugation with D1. 

Finally, the number of D1 peptides per GNRs was evaluated by amino acid analysis, considering the concentration of nanoparticles, determining that, for each gold nanorod, there were 605 ± 95 peptide molecules. It is crucial to determine the orientation of the peptide molecules on the gold surface for the interaction with Aβ. For that reason, we carried out a Raman characterization of the free peptide and the conjugate.

### 3.2. Raman Spectroscopy of GNR-PEG-D1

**Figure 5:**
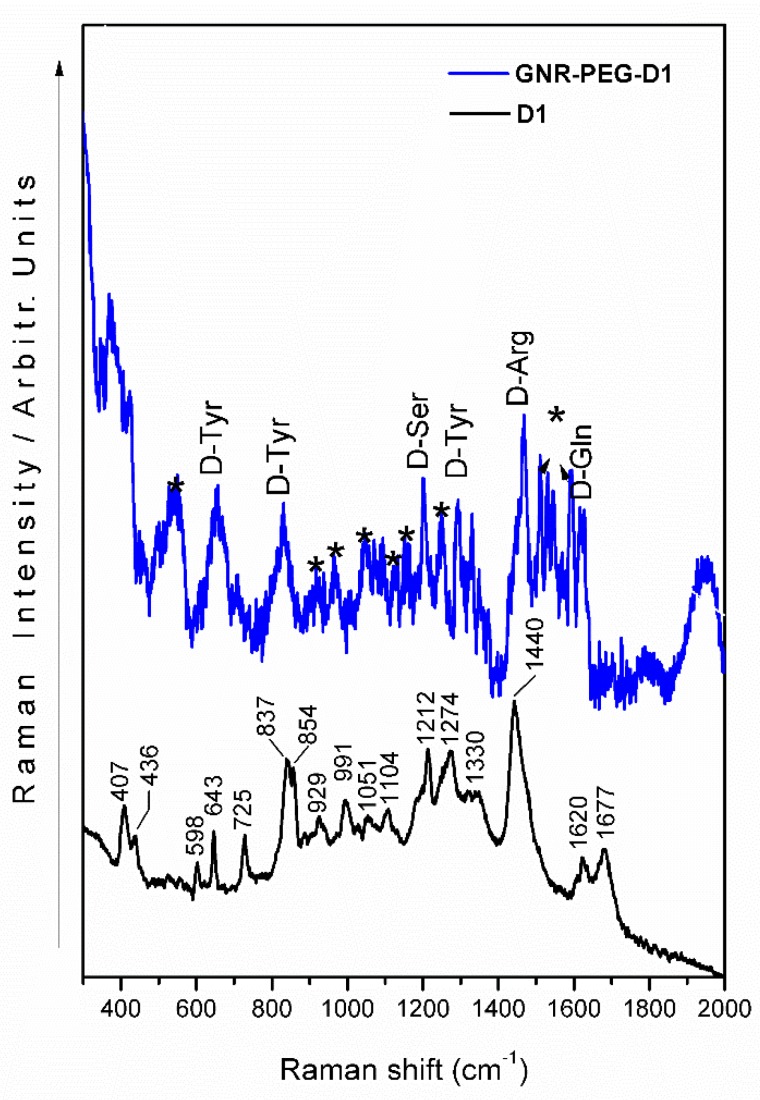
SERS spectrum of the D1 peptide using GNR-PEG-D1 (blue line) and Raman spectrum of D1 peptide (black line). (*: PEG bands).

The Raman spectrum of the peptide D1 exhibited signals with medium-strong relative intensity at 1440, 1274, 1212, 854, 837, 725, 643, 598, and 407 cm^−1^ (Figure 5, black line). The proposed bands assignment, summarized in Table 1, is based on pertinent published data [22,23,24,25,26,27,28]. The strong band at 1440 cm^−1^ is assigned to vibrations related to the guanidinium group of arginine (D-Arg). 

Other bands associated with this amino acid are observed at 1051, 991, and 929 cm^−1^, with medium-weak intensity. The strong band at 837 cm^−1^ is ascribed to a CCN vibration of tyrosine (D-Tyr), which is also accompanied by two signals at 1274 and 643 cm^−1^, assigned to CH and COH deformations, respectively. A medium band at 854 cm^−1^ is due to a coupled vibration of tyrosine and alanine (D-Ala). Alanine also displays a weak band at 598 cm^−1^, which is assigned to the carboxylate group. On the other hand, the presence of glutamine (D-Gln) is inferred from the characteristic bands at 1677 and 1620 cm^−1^, while histidine (D-Hys) is proposed due to its signature band at 725 cm^−1^. The band at 407 cm^–1^ could be originated from deformation modes involving the CN structural moiety.

The SERS spectrum of the system GNR-PEG-D1 (Figure 5, blue line) shows bands ascribed to some amino acid components of the D1 peptide. The most significant peptide signals (D-Tyr, D-Ser, D-Arg, and D-Gln) are observed at 1621, 1470, 1298, 1204, 833, and 658 cm^−1^. Bands observed at 381, 1595, 1576, 1549, 1533, 1515, 1372, 1253, 1163, 1131, 1044, 974, 922, 710, 545, 500, and 423 cm^−1^, highlighted by an asterisk, belong to the PEG molecular component. 

Characteristic SERS amino acid bands of tyrosine (D-Tyr), serine (D-Ser), arginine (D-Arg), and glutamine (D-Gln) were observed. Two medium-broad bands at 658 and 833 cm^−1^ as well as a weak band at 1298 cm^−1^ are assigned to tyrosine vibrations. The signals present at 1204, 1470, and 1621 cm^–1^ are assigned to the aliphatic, guanidinium, and amide fragments of serine, arginine, and glutamine, respectively (Table 1). 

**Figure 6:**
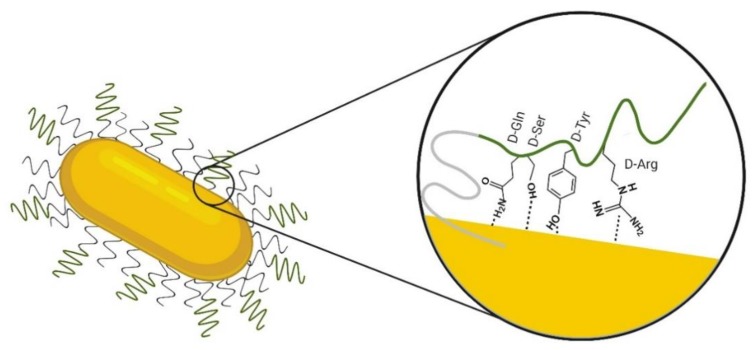
Scheme of the hypothesized orientation of D1(D-Gln-D-Ser-D-hys-D-Tyr-D-arg-D-hys-D-Ile-D-Ser-D-Pro-D-Ala-D-Asn-D-Val) on the GNR-PEG-D1 surface based on SERS results; D1 sequence is represented in a green line and polyethyleneglycol (PEG) in a gray line. Note that we intended to represent the approaching of the different residues to the gold surface. However, we cannot derive a conclusion about the secondary structure of the peptide.

Based on the observed spectral behavior of D1 in the GNR-PEG-D1, the relative intensity of the D-Gln, D-Arg, D-Ser, and D-Tyr bands increase due to surface effect, and the SERS phenomenon rules. As we proposed in [15] where the D1 peptide binds tilted onto the surface of the modified gold nanorod. The wave number shift observed for several amino acid bands suggests that the analyte–metal surface interaction is nearly electrostatic (Figure 6). Additionally, the PEG SERS signals are strong, suggesting a direct interaction of this component with the surface of GNRs. 

### 3.3. Fluorescence In Vitro Assay

**Figure 7:**
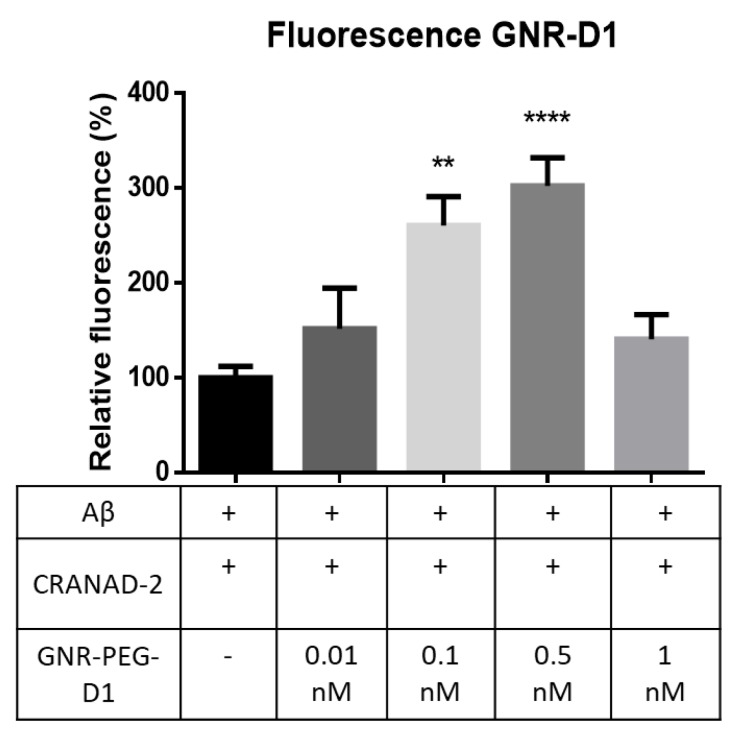
Increase in fluorescence intensity for samples obtained by using CRANAD-2 with GNR-PEG-D1 of different concentrations, at a wavelength of 640 nm and 715 nm. Error bars represent SEM, *n* = 3; *t*-test was used for the statistical analysis to compare baseline values with those obtained values. ** *p* = 0.0011 and **** *p* < 0.0001.

To evaluate the SEF effect due to the presence of GNR-PEG-D1, a 10 µM of Aβ solution was incubated in PBS for 72 h at 37 °C, with constant agitation (300 rpm). Four samples were prepared by adding to this solution CRANAD-2 (1 µM) and GNR-PEG-D1 (in concentrations of 0.01, 0.1, 0.5, and 1 nM). A control sample was also prepared by treating the Aβ solution only with CRANAD-2. The emitted fluorescence at a wavelength of 715 nm (excitation at 640 nm) was measured for all five samples and the relative fluorescence intensity of each sample with respect to the control sample was calculated (fluorescence scan in supplemental material, Figure S5). The results, presented in Figure 7, indicate an increase in fluorescence intensity by a factor of 2.5 (*p* = 0.0011) and 3 (*p* < 0.0001) for the samples with 0.1 and 0.5 nM GNR-PEG-D1, respectively. The addition of GNR-PEG-D1 produced an increase in the fluorescence of the CRANAD-2 probe bound to the Aβ fibrils, demonstrating that this is the system that produces the SEF effect. A lesser fluorescence enhancement was observed at higher GNR-PEG-D1 concentrations (1 nM), possibly due to a quenching effect produced by the plasmonic nanoparticles [29]. The concentration of CRANAD-2, nanoparticles, and Aβ is critical to determine the density of the components and the distance between them to obtain the SEF effect. To have an efficient SEF effect a minimal distance between the fluorescent probe and the surface of the nanoparticle is necessary to avoid fluorescence quenching [30]. The process to enhance the fluorescent emission by taking into account the proximity factor is labeled by an electromagnetic mechanism. Otherwise, when the fluorophore and the nanoparticle are interacting through a chemical mechanism (by a bond or pseudo-bond), the quenching of fluorescence is due to energy transfer from the analyte to the metal surface via a nonradiative pathway [30]. Finally, this energy is emitted to the surroundings via nanoparticle phonons.

At low concentrations of either GNRs or CRANAD-2, the probability of getting the proper distance decreases. On the other hand, a higher than optimal concentration could imply that the proximity between GNRs and CRANAD-2 could be shorter than optimal, producing a quenching effect attributed to the short distance between CRANAD-2 and the nanoparticles. The distances between the fluorophore, the nanoparticle, and the Aβ aggregates will be evaluated in future studies.

On the other hand, GNRs-PEG does not produce an enhancement of the fluorescence intensity of Aβ aggregates present in the slides in the presence of CRANAD-2 (Supplementary Material, Figure S4). This can be explained due to GNRs-PEG not containing the recognizing element of Aβ that is the D1 peptide. A low degree of interaction of GNRs-PEG with Aβ is expected due to the PEG nanoparticle´s surface coatings being widely used for their ability to reduce protein adsorption (in this case with Aβ), to diminish non-specific interactions with cells, and to improve pharmacokinetics [31]. 

**Figure 8:**
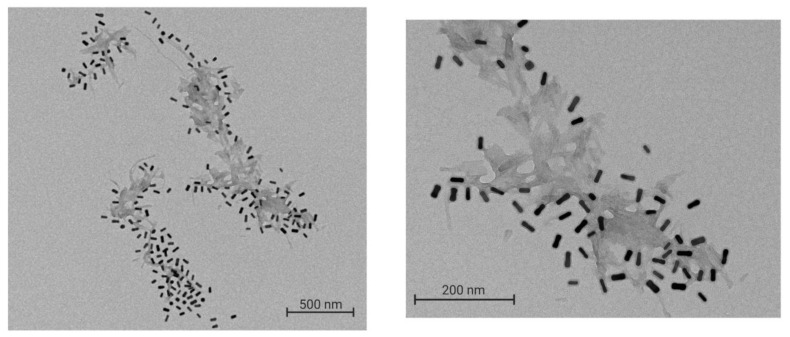
TEM image of GNR-PEG-D1 decorating the Aβ fibrils. A total of 0.5 nM GNR-PEG-D1 was incubated with a 10 μM Aβ solution for 20 min at 37 °C, with mechanic agitation. Left image: magnification x 10,000; right image: magnification x 30,000.

The interaction between the Aβ fibrils and nanoparticles was observed by TEM. The TEM images obtained clearly show the localization of GNR-PEG-D1 in the vicinity of the fibrils (Figure 8).

### 3.4. Fluorescence Ex Vivo in Histological Sections of Alzheimer’s Disease Model

In order to probe the effect of GNR-PEG-D1 on biological samples, we evaluated the SEF effect on the brain tissue of AD mice that exhibited amyloid plaques. The fluorescence signals emitted by CRANAD-2 in the presence and in the absence of nanoparticles were compared ex vivo by histological analysis. Histological slices of a transgenic AD model were collected from APPswe/PSEN1dE9 mice aged one year and seven months when the amyloid plaques can be clearly observed by fluorescence microscopy. The histological sections were incubated with 0.24 mM CRANAD-2, subsequently treated with GNR-PEG-D1, and then evaluated to determine the fluorescent signal. The slices from the same brain regions were used for all samples measured.

A screening of different concentrations of GNR-PEG-D1 was carried out in order to obtain the optimal concentration needed for enhancing the fluorescence signal. Five concentrations of GNR-PEG-D1 (0.5, 0.1, 0.01, 0.001, and 0.0001 nM), were incubated with the brain tissues (Supplementary Material, Figure S6). For higher concentrations (0.5, 0.1, and 0.01 nM), no significant increase in the fluorescent signal was observed, possibly due to a quenching effect described for GNPs and others plasmonic and/or metallic nanoparticles [29,32]. However, the GNR-PEG-D1 at 0.001 nM showed an effective increase in the fluorescence emitted by CRANAD-2 due to the SEF effect. Lower concentration of GNR-PEG-D1, 0.0001 nM, did not show an increase in the fluorescent signal of CRANAD-2, probably because the nanoparticle solution was too diluted (Supplementary Material, Figure S6), indicating that a critical minimal concentration of GNRs is needed to produce the SEF effect in the system tested. As the distance between the gold nanoparticle´s surface and the fluorescent probe is very relevant for the SEF effect [33,34], this distance depended on the disposition of GNRs and CRANAD-2 over the amyloid fibers on the tissue in this case. 

**Figure 9:**
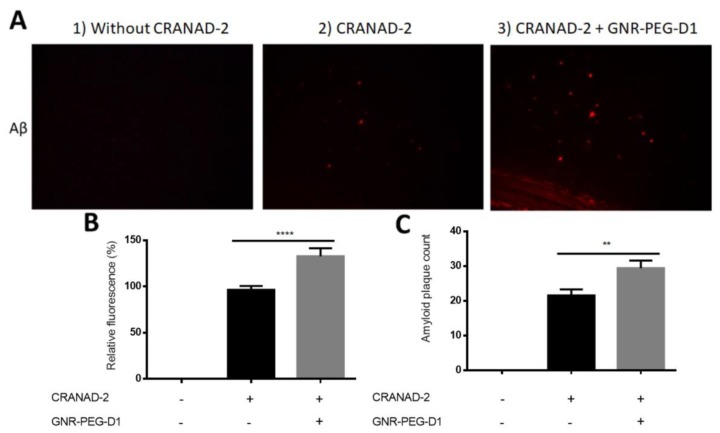
GNR-PEG-D1 presence enhances the fluorescent signal emitted by CRANAD-2. (**A**) Images of cortex histological sections of the Alzheimer’s disease (AD) model. From left to right: brain tissue prior to any treatment (1), incubated with CRANAD-2 0.24 mM for 5 min and washed with ethanol (2), and incubated with GNR-PEG-D1 0.001 nM for 5 min (3). (**B**) Quantitative and statistical analysis of the total number of images (n). (**C**) Count of amyloid plaques for the total number of images. Error bars represent SEM, *n* = 39. Mann–Whitney test was used for the statistical analysis. ** *p* = 0.0011, **** *p* < 0.0001.

Images of the histological specimens of the cerebral cortex and the corresponding relative fluorescence are presented in Figure 9. In the upper panel of Figure 9, the baseline fluorescence signal of the brain tissue incubated with CRANAD-2 is clearly observed, as well as the fluorescence enhancement after incubation with both CRANAD-2 and GNR-PEG-D1 (Figure 9A). In the lower panel of Figure 9, the results for the relative intensity of the fluorescent signal emitted by CRANAD-2 (Figure 9B) and the count of amyloid plaques (Figure 9C) are presented, with and without incubation with GNR-PEG-D1. When tissues were incubated with GNR-PEG-D1, the amyloid plaque count increased to 1.4 ± 0.5 times (*p* = 0.0058), and the relative fluorescence, 1.4 ± 0.1 times (*p* < 0.0001). These results indicate that the GNPs produce an enhancement in the fluorescence signal emitted by CRANAD-2 associated to amyloid plaques. Moreover, a higher number of plaques can be detected in the presence of the conjugate, a very relevant factor to improve the detection of amyloid plaques, which can make the difference between a timely or a late diagnosis, where the disease has already progressed. However, clear CRANAD-2 and GNR-PEG-D1 concentrations are key factors in observing these effects. An excess of these compounds can lead to a quenching phenomenon of the SEF effect [35].

As control, when the tissues were incubated with GNR-PEG, no increase of fluorescence intensity of CRANAD-2 linked to Aβ plaques was observed (Supplementary Material, Figure S7). This could be attributed to a low interaction of the GNRs-PEG with the amyloid plaques or the possible disposition of this nanosystem that can modify the distance between the GNRs and CRANAD-2, impeding the SEF effect. 

## 4. Conclusions

In this research, we developed and evaluated a nanosystem based on GNPs functionalized with D1 peptide which, in the presence of CRANAD-2, increased the fluorescence intensity signal of CRANAD-2 both in vitro and ex vivo. These results evidence a significant surface fluorescence enhancement effect produced by the SPR of GNPs tagging Aβ fibrils, allowing an improvement in their detection by fluorescence. We envisage that our nanosystem co-administered in vivo with a fluorescent NIR probe used for in vivo detection of β-amyloid can increase the fluorescence signal attributed to toxic aggregates, allowing an improvement of imaging, which is crucial for a better AD diagnostic. This technique may be applied for in vivo detection of amyloids, contributing to a more reliable use of fluorescence imaging for AD detection.

## Figures and Tables

**Table 1:** Raman and SERS signals assignment for D1 and GNR-PEG-D1. D-Arg: arginine; D-Tyr: tyrosine; D-Ser: serine; D-Gln: glutamine; D-Ala: alanine; D-His: histidine. ν: stretching. δ: bending. def.: deformation. The stretching mode (ν) is associated with the change in the continuum interatomic length of a couple of bonded atoms, while the bending mode (δ) is related with the change of an angle that is formed by two bonds.

Raman D1 (cm^−1^)	SERS GNR-PEG-D1 (cm^–1^)	Assignment
1677		D-Gln Amide I (νC=O + δNH)
1620	1621	D-Gln
1440	1430	D-Arg
1274	1298	D-Tyr
1212	1204	D-Ser Amide III def.
1051		D-Arg
991		D-Arg
929		D-Arg/D-Gln
854		D-Tyr/D-Ala
837	833	D-Tyr
725		D-His
643	658	D-Tyr
598		D-Ala
436		δCN def.
407		δCN def.

## Supplementary Materials

The following are available online at https://www.mdpi.com/2079-4991/10/4/690/s1, Figure S1. UPLC Peptide D1. Figure S2. Peptide D1 mass spectrum. Table S1. Raman and SERS signals assignment for the D1, PEG and GNR-PEG-D1 systems. Figure S3. Representative image of DLS graphics of GNR-CTAB), GNR-PEGs and GNR-PEG-D1. Figure S4. Fluorescence values obtained for experimental controls. Figure S5. Fluorescence scans obtained from the interaction of Aβ with CRANAD-2 by adding different concentrations of GNR-PEG-D1. Figure S6. Screening of different GNR-PEG-D1 concentrations and their effect on the fluorescent signal of CRANAD-2. Figure S7. Incubation of tissues with CRANAD-2 and GNR-PEG do not show significant differences with respect to the control. Figure S9. Electrospray ionization (ESI)-MS spectrum of Curcuminoid. Figure S10. ^1^H-NMR spectrum of CRANAD-2 in DMSO-d_6_. Figure S11. Electrospray ionization (ESI)-MS spectrum of CRANAD-2. The sample was dissolved in acetonitrile (equipment LCMS-2020). Figure S12. UV-vis absorption spectrum of curcuminoid (red) and CRANAD-2 (black), in CHCl_3_ at 1 x 10^-5^M. Figure S13. Fluorescent emission spectrum of curcuminoid (red) and CRANAD-2 (black) in CHCl_3_ at 1 × 10^−5^M with 1% of Transmittance.
