# Supplementary material for: Gold Nanoparticles Mediate Improved Detection of β-amyloid Aggregates by Fluorescence
Source: Nanomaterials (Basel). 2020 Apr 6;10(4):690. doi: 10.3390/nano10040690 (PMC7221977; doi:10.3390/nano10040690)
Supplement: Supplementary file 1 [file nanomaterials-10-00690-s001.pdf]

Supplementary Materials

Gold Nanoparticles Mediate Improved Detection of β-amyloid Aggregates by Fluorescence

Pedro Jara-Guajardo [1,2], Pablo Cabrera [1,2], Freddy Celis [3], Mónica Soler [4], Isadora Berlanga [4], Nicole Parra-Muñoz [4], Gerardo Acosta [5], Fernando Albericio [5,6], Fanny Guzman [7], Marcelo Campos [8], Alejandra Alvarez [9,10] and Francisco Morales-Zavala [1,2,*],Marcelo J Kogan [1,2,*].

[1] Departamento de Química Farmacológica y Toxicológica, Facultad de Ciencias Químicas y Farmacéuticas, Universidad de Chile, Santiago 8380494, Chile; pedro.jaraguajardo@gmail.com (P.J.-C.); pablo.cabrera@ug.uchile.cl (P.C.)
[2] Advanced Center for Chronic Diseases (ACCDiS), Sergio Livingstone 1007, Independencia, Santiago 8380494, Chile
[3] Laboratorio de Procesos Fotónicos y Electroquímicos, Universidad de Playa Ancha, Valparaíso, Chile; freddy.celis.b@gmail.com
[4] Departamento de Ingeniería Química, Biotecnología y Materiales, Facultad de Ciencias Físicas y Matemáticas, Universidad de Chile, Beaucheff 851, Santiago 8380494, Chile; m.soler.jauma@gmail.com (M.S.); isadora.berlanga@ing.uchile.cl (I.B.); nparra@ing.uchile.cl (N.P.-M.)
[5] CIBER-BBN, Networking Centre on Bioengineering, Biomaterials and Nanomedicine & Department of Organic Chemistry, Marti i Franques 1-11, University of Barcelona (UB), 08028 Barcelona, Spain; gerardoacosta@ub.edu (G.A.); albericio@ub.edu (F.A.)
[6] School of Chemistry & Physics, University of KwaZulu-Natal, Durban 4001, South Africa
[7] Núcleo de Biotecnología Curauma (NBC), Pontificia Universidad Católica de Valparaíso, Valparaíso 2460355, Chile; fanny.guzman@pucv.cl
[8] Department of Chemistry, Faculty of Sciences, University of Chile, POBox 653, Santiago 8380494, Chile; facien05@uchile.cl
[9] Facultad de Ciencias Biológicas, Pontificia Universidad Católica de Chile, Alameda 340, Santiago 8331010, Chile; aalvarez@bio.puc.cl
[10] Centro de envejecimiento y regeneración (CARE), Facultad de Ciencias Biológicas, Pontificia Universidad Católica de Chile, Santiago 8380494, Chile
* Correspondence: fmorales@ciq.uchile.cl (F.M.-Z.); mkogan@ciq.uchile.cl (M.J.K.)

Peptide D1 (H-qshyrhispaqv-OH), was synthesized with all amino acids with D configuration, that increases the stability in from of proteases.

The synthesis was carried out using the HMPB Chem Matrix resin the loading 0.49 mmol/g, that allows us to have peptides with the carboxylic acid in the C-Terminal, and is ideal for this type of peptide with so many branched amino acids, due to the flexibility provided by the polyethylene glycol polymer.

0.3 g of resin are conditioned by washing with DMF, DCM, DMF, MeOH, DMF and DCM twice with each one for 1 minute and stirring with a Teflon rod. Once the resin is swollen, the first amino acid la Val is coupled by the symmetric anhydride method using (10 equiv, 0.50 mg) of Val, (5 equiv, 120 L) of DIC (Diisopropylcarbodiimide) and (0.1 equiv, 0.002 mg) of DMAP (Dimethylaminopyridine) in DCM anhydrou. Since the Val is -branched amino acid, two treatments are made, one of 4 hours and another overnight to ensure complete incorporation.

The elongation of the peptide chain was done using an Fmoc / tBu strategy using 3 equiv AA, 3 equiv Oxyma as additive and 3 equiv DIC as coupling agent in DMF for 45–60 min. Deprotection of the Fmoc group with 20% Piperidine in DMF with 2 washes of 5 min each.

The resin was cleaved by acidolysis with a mixture (TFA-H_2O-Tis) in a ratio (95: 2.5: 2.5) for two hours, after this time the acidic solution of the peptide was added over cold diethyl ether for precipitation. The solid is decanted and washed again with cold diethyl ether. The solid obtained, 150 Mg 71.77% yield, is dissolved in a mixture of H_2O / ACN (80:20) and lyophilized.

The peptide was purified in a semi-preparative HPLC with built-in mass detector using a 5–50% gradient of ACN in 10 min at 25 °C and collected by masses, Column: XBridge BEH130 C18 5 m, 50 x 100 mm; eluents: water with 0.1% TFA and ACN with 0.1% TFA, detection at 214 nm. 89.4 mg was obtained with a 59.6% recovery. The peptide D1 was characterized by HPLC and HPLC-MS. As it is shown in Figures S2 and S3

Peptide D1 with retention time 1121 and 100% purity in a gradient 0-100 in 2 minutes. Mass calculated 1422.57, Mass found: 1423.5 $[M+H]^{+1}$; 712.5 $[M+2H]^{+2}$; 475.1 $[M+3H]^{+3}$; 356.6 $[M+4H]^{+4}$.

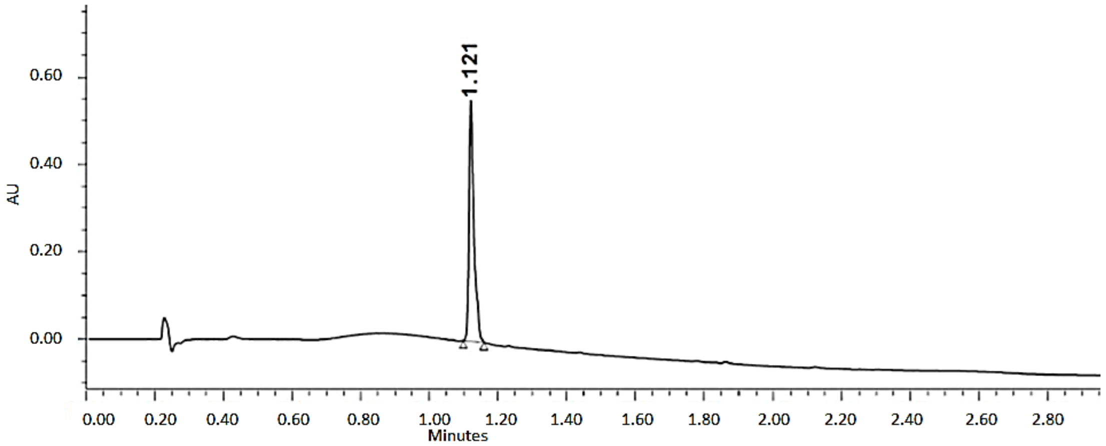

Figure S1. UPLC Peptide D1 Column: BEH C8 1,7 m 2.1 × 50 mm, gradient 0–100% ACN time 2 min, temperature 40 °C. eluents water 0.045% of TFA and CAN 0.036% of TFA. Retention time 1.121 min.

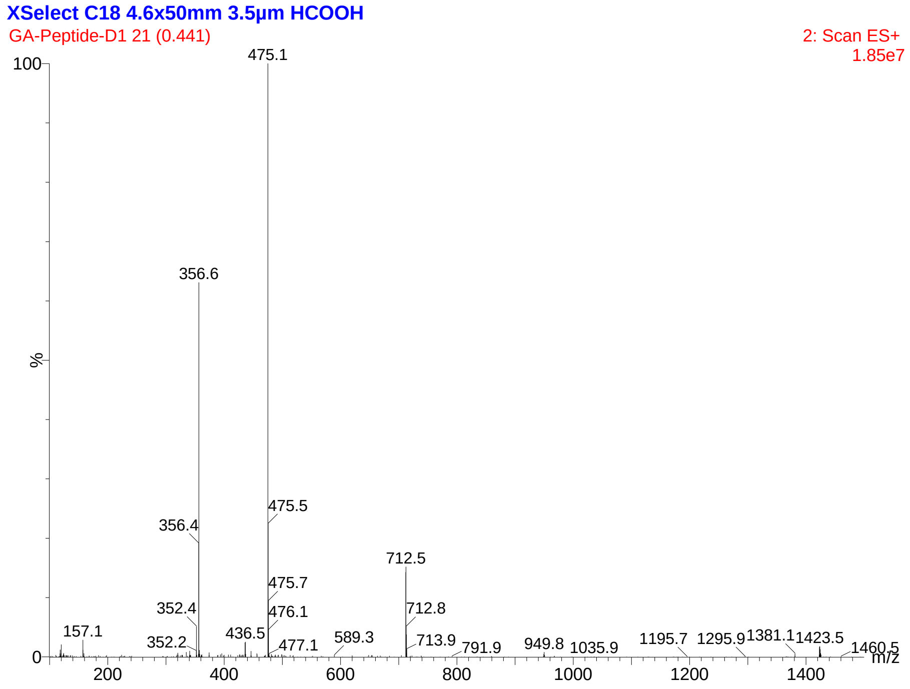

Figure S2. Peptide D1 mass spectrum. HPLC-MS Column: XSelect C18 3.5 m 4.6 × 50 mm. Eluent: H_2O with 0.1% formic acid and ACN with 0.07% formic acid Gradient: 0–50%ACN in 3.5 min. Mass calculated 1422.57, Mass found: 1423.5 $[M+H]^{+1}$; 712.5 $[M+2H]^{+2}$; 475.1 $[M+3H]^{+3}$; 356.6 $[M+4H]^{+4}$.

Table S1. Raman and SERS signals assignment for the D1, PEG and GNR-PEG-D1 systems. The stretching mode (ν) is associated with the change in the continuum interatomic length of a couple of bonded atoms, while the bending mode (δ) is related with the change of an angle that is formed by two bonds.

Raman D1 (cm^{-1})	SERS<br>GNR-PEG-D1<br>(cm^{-1})	Assignment
1677 m		D-Gln<br>Amide I ($\nu C{=}O + \delta NH$)
1620 m	1621 s	D-Gln +<br>PEG vib<br>OCH_2 def.
	**1595 s**	PEG vib<br>OCH_2 def.
	**1576 w**<br>**1549 m**<br>**1533 m**	PEG vib<br>OCH_2 def.
	**1515 m**	PEG vib<br>OCH_2 def.
1440 vs	1470 s	D-Arg
	**1372 w**	PEG vib<br>$\varpi(CH_2)_i + \nu CC$
1274 s,br	1298 m,s	D-Tyr
	**1253 m,w**	PEG vib<br>$t(CH_2)_{i+o}$
1212 s	1204 m,s	D-Ser<br>Amide III def.
	**1163 w**<br>**1131 w**	PEG vib<br>$\nu(CO)_{o+i} + r(CH_2)_i$
1051 w		D-Arg
	**1044 m**	PEG vib<br>$\nu(CO)_i + r(CH_2)_i$
991 m		D-Arg
	**974 w,br**	PEG vib<br>$\nu(CO)_i + r(CH_2)_i$
929 w		D-Arg/D-Gln
	**922 w**	PEG vib
		PEG vib<br>$\nu(CO)_i + r(CH_2)_i$
854 s		D-Tyr/D-Ala
837 s	833 s,br	D-Tyr
		PEG vib<br>$r(CH_2)_i$
		PEG vib<br>$r(CH_2)_i$
725 m		D-His
	**710 w**	PEG vib<br>$\delta(OCC)o$
643 m	658 s,br	D-Tyr +<br>PEG vib<br>$\delta(OCC)o$
598 m,w		D-Ala +<br>PEG vib<br>$\delta(OCC)o$
	**545 s,br**	PEG vib<br>$\delta(OCC)o$

	**500 w**	PEG vib<br>δ(OCC)o
436 m,w		δCN
	**423 sh**	PEG vib<br>δ(OCC)$_{o+i}$
407 s		δCN
	**381 s**	PEG vib<br>δ(OCC)$_{o+i}$
D-Arg: arginine; D-Tyr: tyrosine; D-Ser: serine; D-Gln: glutamine; D-Ala: alanine; D-His: histidine.; def.: deformation; vib: vibration; br: broad; w: weak; m: medium; s: strong; v: very; sh: shoulder; i: in-plane; o: out-of-plane; t: twisting; ϖ: wagging; δ: bending; ν: stretching; r: rocking;		

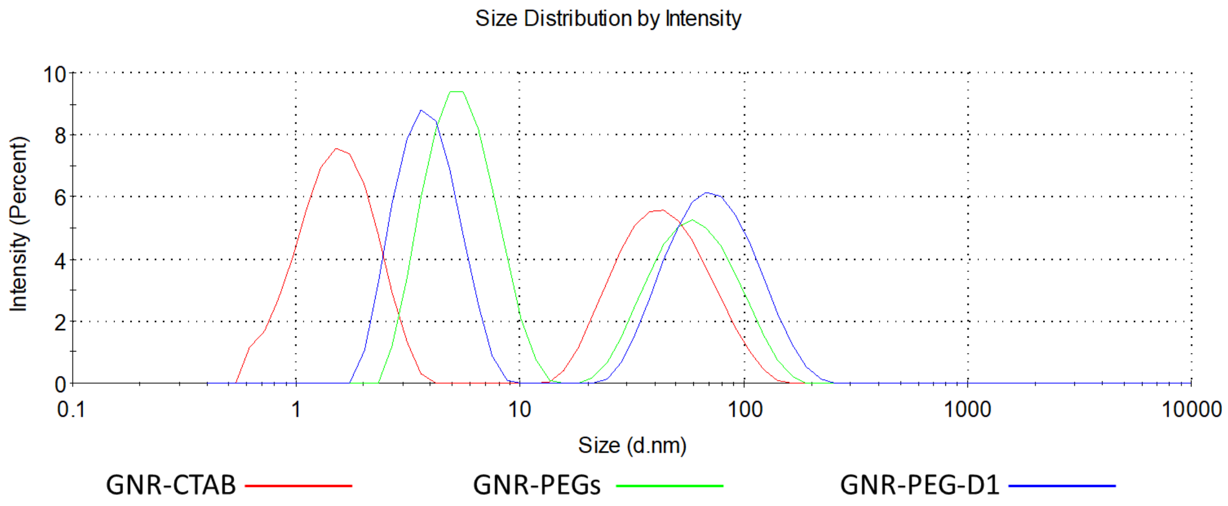

Figure S3. Representative image of DLS graphics. GNR-CTAB (red line), GNR-PEGs (green line) and GNR-PEG-D1 (blue line).

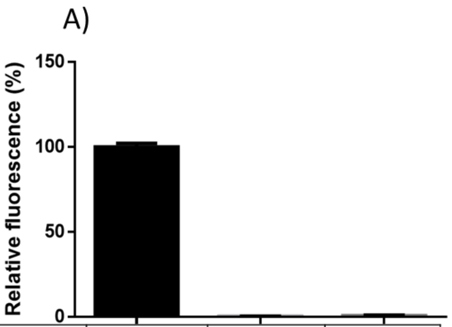

Aβ	+	+	-
CRANAD-2	+	-	+
GNR-PEG-D1	-	-	-

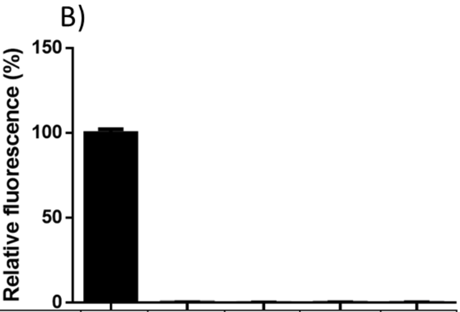

Aβ	+	-	-	-	-
CRANAD-2	+	-	-	-	-
GNR-PEG-D1	-	0.01 nM	0.1 nM	0.5 nM	1 nM

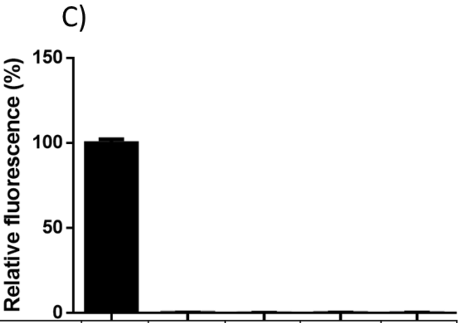

Aβ	+	-	-	-	-
CRANAD-2	+	+	+	+	+
GNR-PEG-D1	-	0.01 nM	0.1 nM	0.5 nM	1 nM

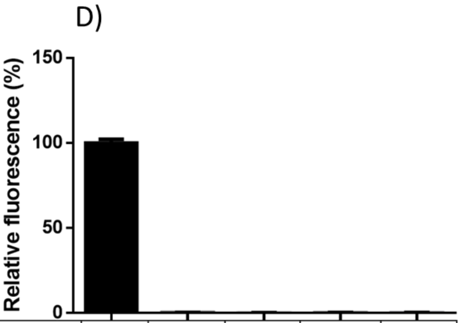

Aβ	+	+	+	+	+
CRANAD-2	+	-	-	-	-
GNR-PEG-D1	-	0.01 nM	0.1 nM	0.5 nM	1 nM

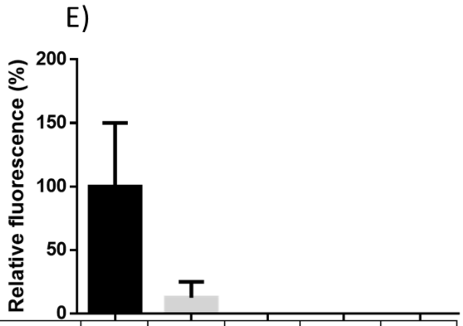

Aβ	+	+	+	+	+
CRANAD-2	+	+	+	+	+
GNR-PEG	-	0.01 nM	0.1 nM	0.5 nM	1 nM

Figure S4. Fluorescence values obtained for experimental controls. Aβ fibrils were obtained using the methodology outlined in the methodology section, once incubated, fluorescence studies were carried out to obtain fluorescence values for Aβ aggregates alone, with GNR-PEG-D1 or with GNRs-PEG. In addition, the fluorescence of CRANAD-2 alone and GNR-PEG-D1 alone were evaluated. A) Basal fluorescence control of Aβ alone and CRANAD-2 alone. B) Basal Fluorescence control of GNR-PEG-D1 (0.01; 0.1; 0.5 and 1 nM). C) Basal Fluorescence control of GNR-PEG-D1 (0.01; 0.1; 0.5 and 1 nM) with CRANAD-2. D) Basal fluorescence control of GNR-PEG-D1 (0.01; 0.1; 0.5 and 1 nM) with Aβ. E) Basal fluorescence control of GNR-PEG with CRANAD-2 and Aβ. The fluorescence of all samples was measured with an excitation wavelength of 640 nm and an emission wavelength of 715 nm.

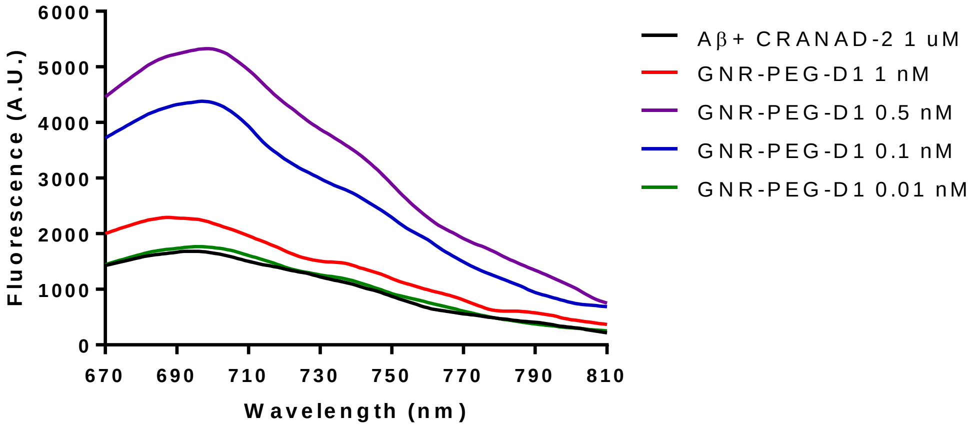

Figure S5. Fluorescence scans obtained from the interaction of Aβ with CRANAD-2 (black line) and by adding different concentrations of GNR-PEG-D1 to the mixture of Aβ and CRANAD-2 (0.01nM green line; 0.1 nM blue line; 0.5 nM purple line; 1 nM red line). An excitation wavelength of 640 nm was used and for the emission scan a range of wavelengths from 670 nm to 810 nm was used.

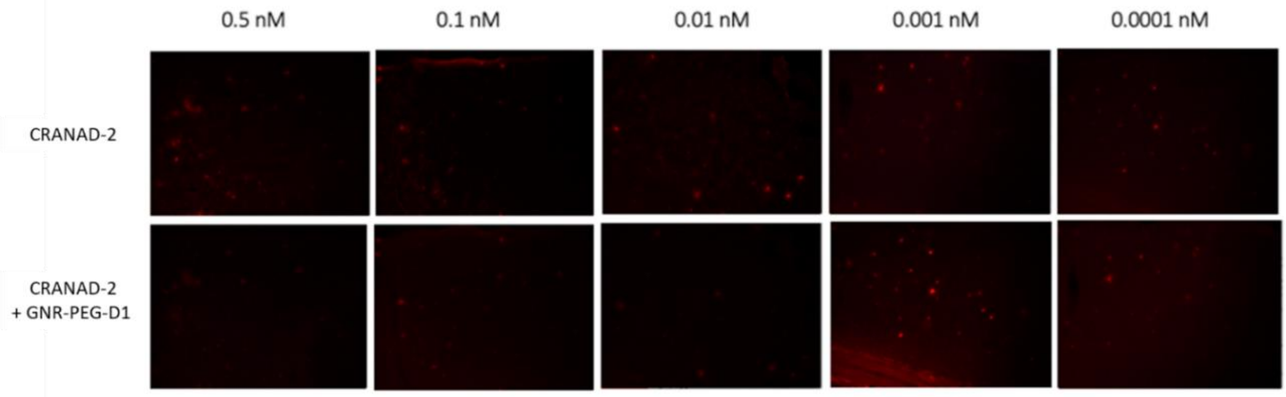

Figure S6. Screening of different GNR-PEG-D1 concentrations and their effect on the fluorescent signal of CRANAD-2. The upper panel shows images corresponding to cerebral cortex histological sections of the AD model, treated under different conditions. All images were taken after treatment with CRANAD-2, and without GNR-PEG-D1. The lower panel shows the same histological sections used for the images presented in the upper panel, after treatment with GNR-PEG-D1 in different concentrations. Histological sections previously incubated with CRANAD-2 0.24 mM for 5 min and then washed with the ETOH battery, were subsequently treated with 0.5, 0.1, 0.01, 0.001 and 0.0001 nM GNR-PEG-D1 for 5 min, before taking the pictures.

A

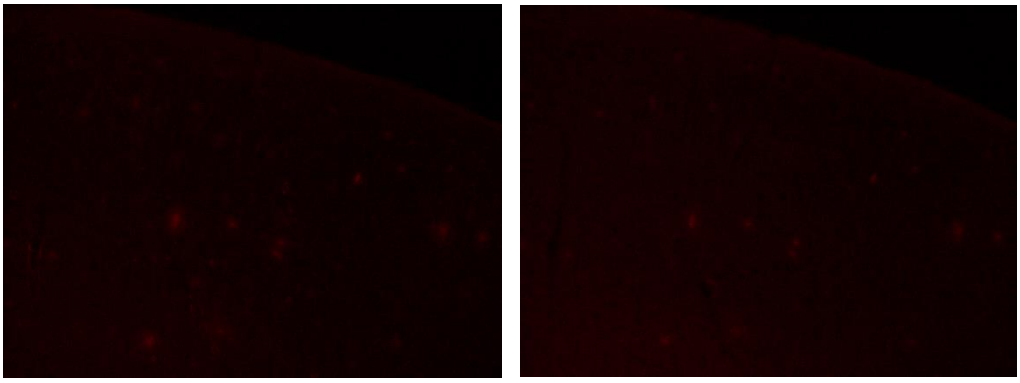

CRANAD-2 | CRANAD-2 + GNR-PEG

B

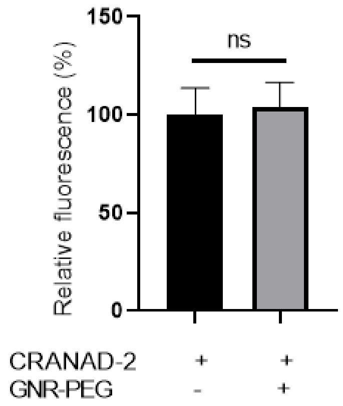

C

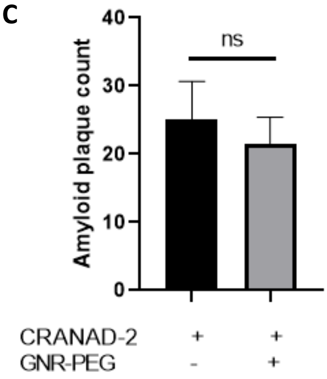

Figure S7. Incubation of tissues with CRANAD-2 and GNR-PEG do not show significant differences with respect to the control. A) Panels A show images corresponding to cerebral cortex histological sections of the AD model, treated under different conditions. B) Relative fluorescence intensity (%) ± SEM of n = 11 with respect to the sample without nanoparticles. (C) Graph shows the count of the number of plates of the total images obtained. (Error bars represent SEM, n = 11). Mann-Whitney test was used for the statistical analysis. **** p <0.0001.

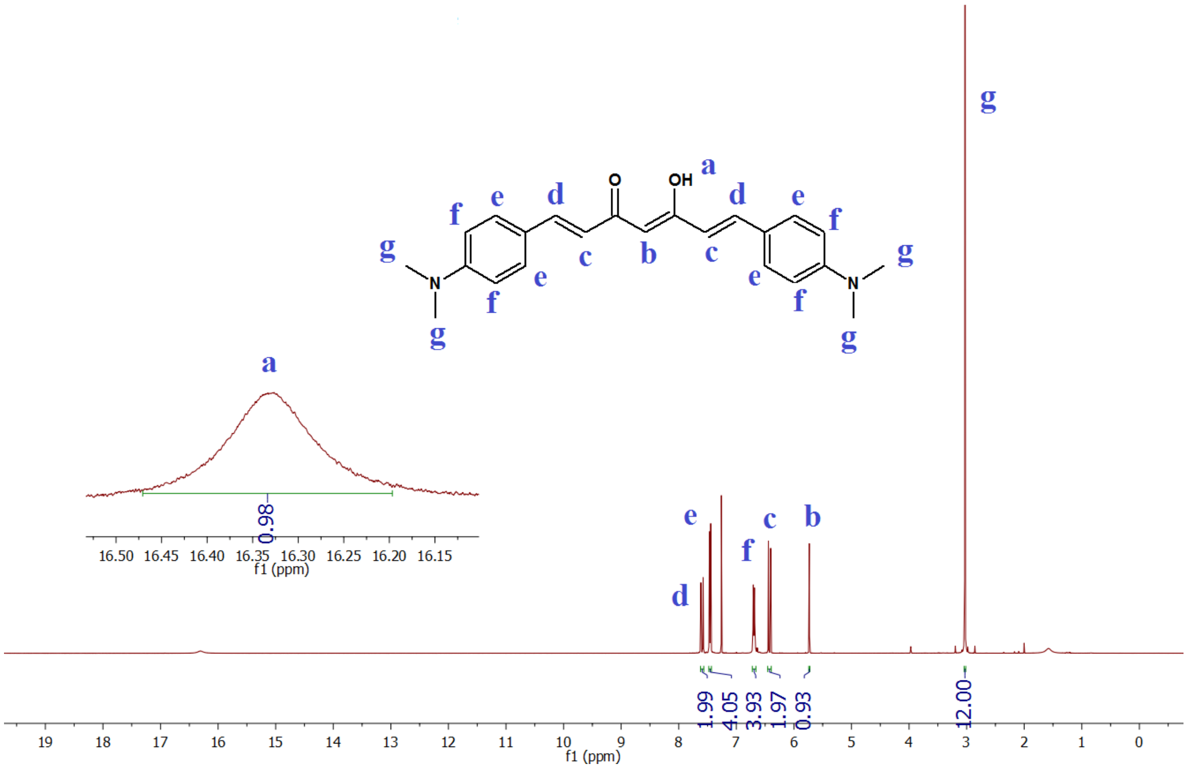

Figure S8. ^{1}H-NMR spectrum of Curcuminoid in $CDCl_3$.

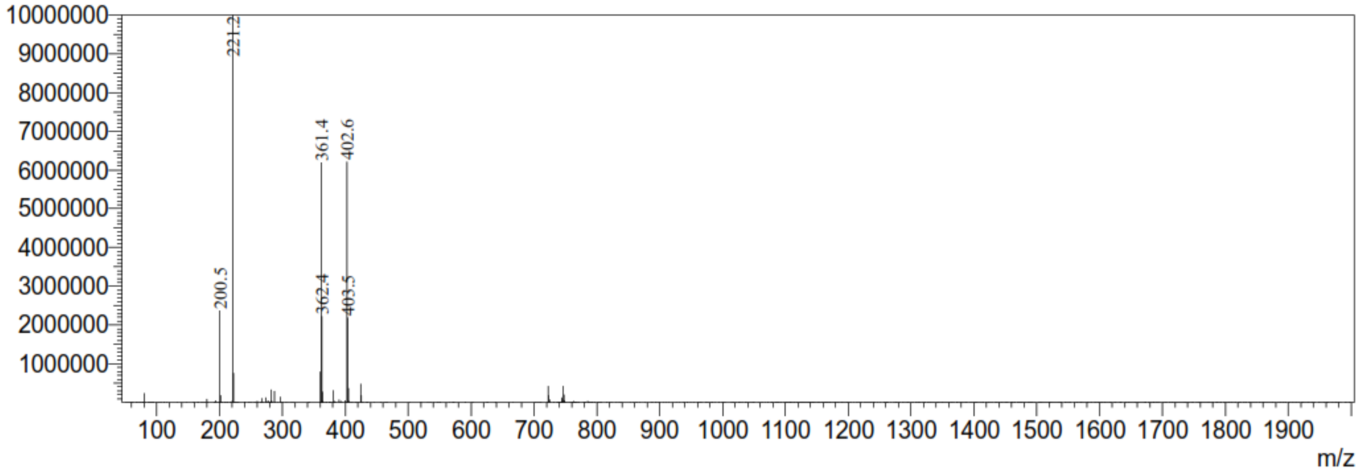

Figure S9. Electrospray ionization (ESI)-MS spectrum of Curcuminoid. The sample was dissolved in acetonitrile (equipment LCMS-2020).

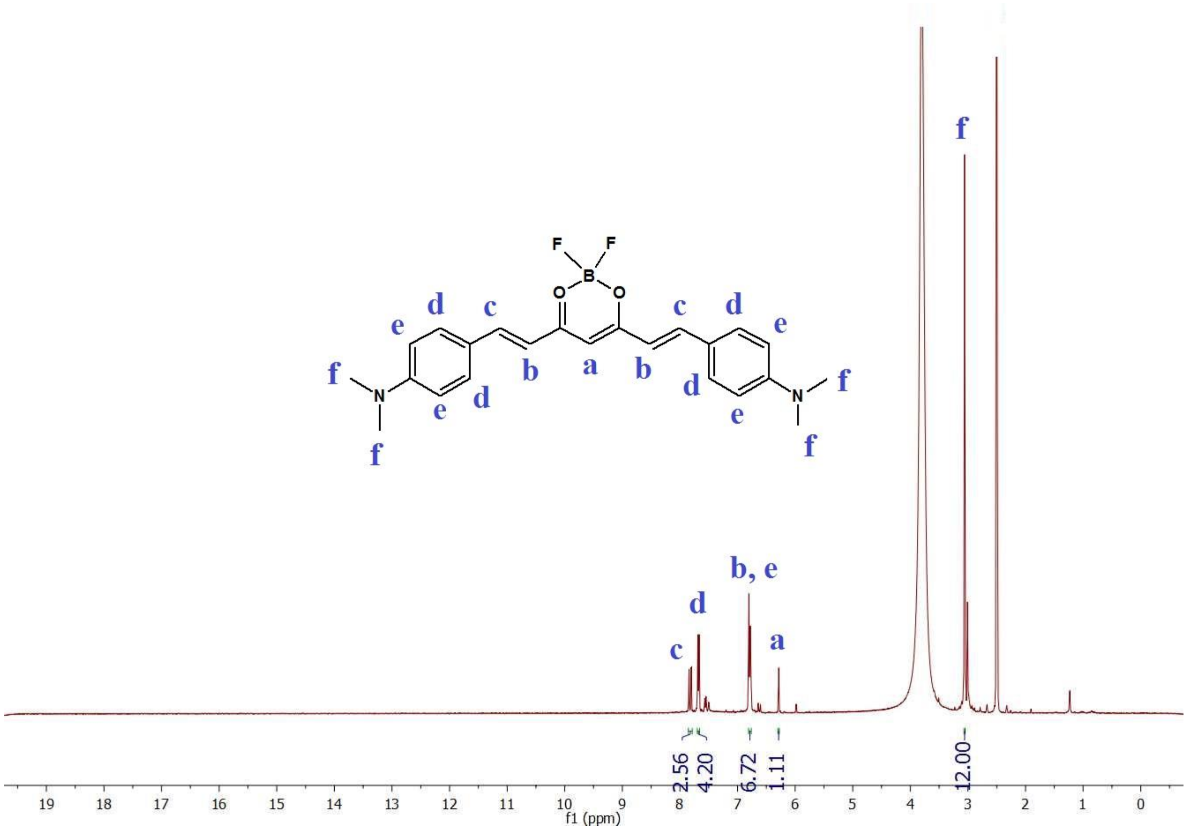

Figure S10. ^{1}H-NMR spectrum of CRANAD-2 in DMSO-d_6.

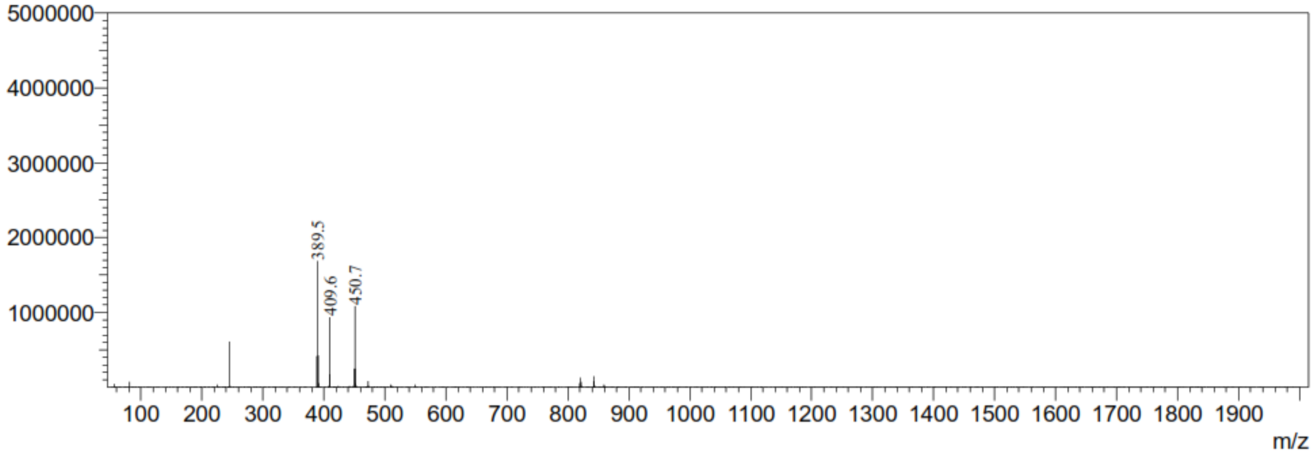

Figure S11. Electrospray ionization (ESI)-MS spectrum of CRANAD-2. The sample was dissolved in acetonitrile (equipment LCMS-2020).

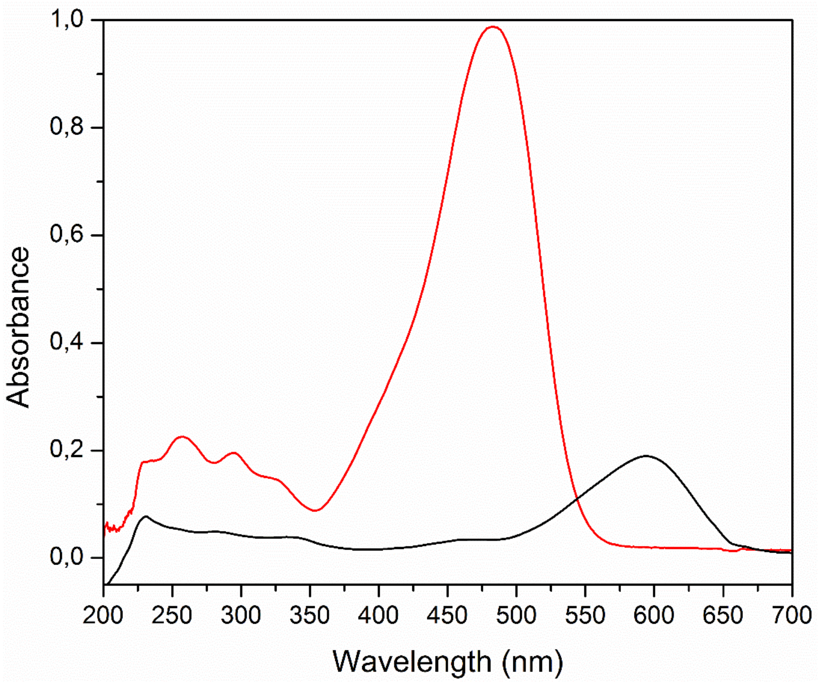

Figure S12. UV-vis absorption spectrum of curcuminoid (red) and CRANAD-2 (black), in $CHCl_3$ at 1 x 10^{-5}M.

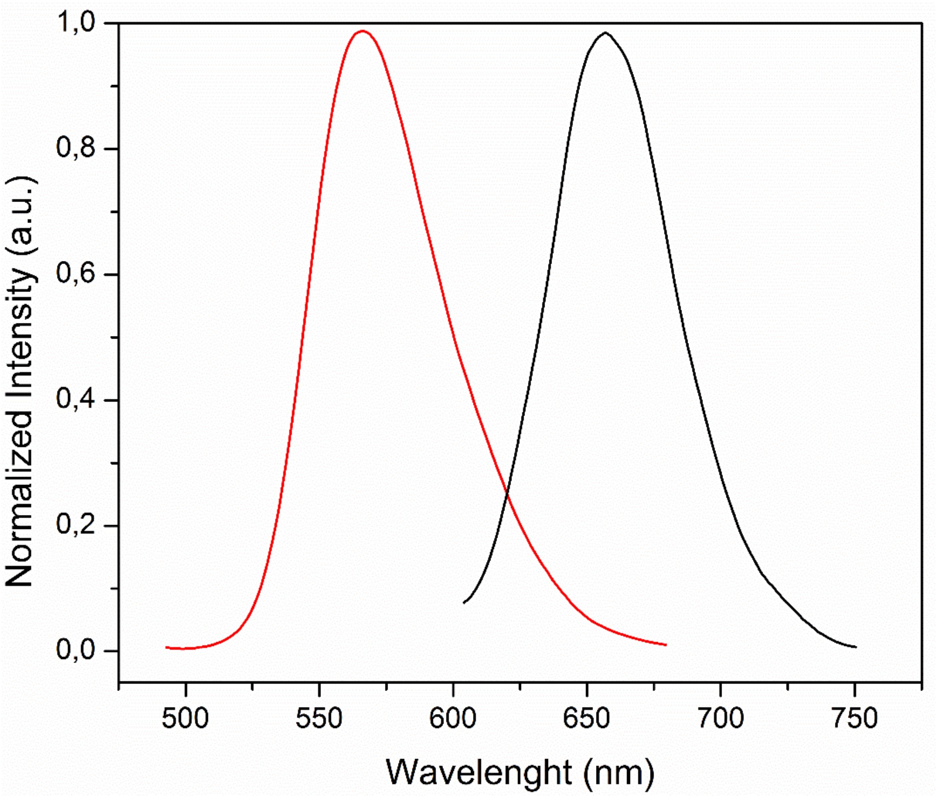

Figure S13. Fluorescent emission spectrum of curcuminoid (red) and CRANAD-2 (black) in $CHCl_3$ at 1 × 10^{-5}M with 1% of Transmittance.
